# Supplementary material for: AVOCADO: Visualization of Workflow–Derived Data Provenance for Reproducible Biomedical Research
Source: Comput Graph Forum. Author manuscript; Available in PMC 2018 Jul 2. (PMC6027754; doi:10.1111/cgf.12924)
Supplement: Supplementary Information [file NIHMS835059-supplement-Supplementary_Information.pdf]

Eurographics Conference on Visualization (EuroVis) 2016
K.-L. Ma, G. Santucci, and J. van Wijk
(Guest Editors)

Volume 35 (2016), Number 3

Supplement - AVOCADO: Visualization of Workflow-Derived Data Provenance for Reproducible Biomedical Research

H. Stitz[1], S. Luger[1], M. Streit[1]*, and N. Gehlenborg[2]*

[1]Johannes Kepler University Linz, Austria
[2]Harvard Medical School, United States of America
*Equal contribution

1. Usage Scenario: Additional Figures and Workflows

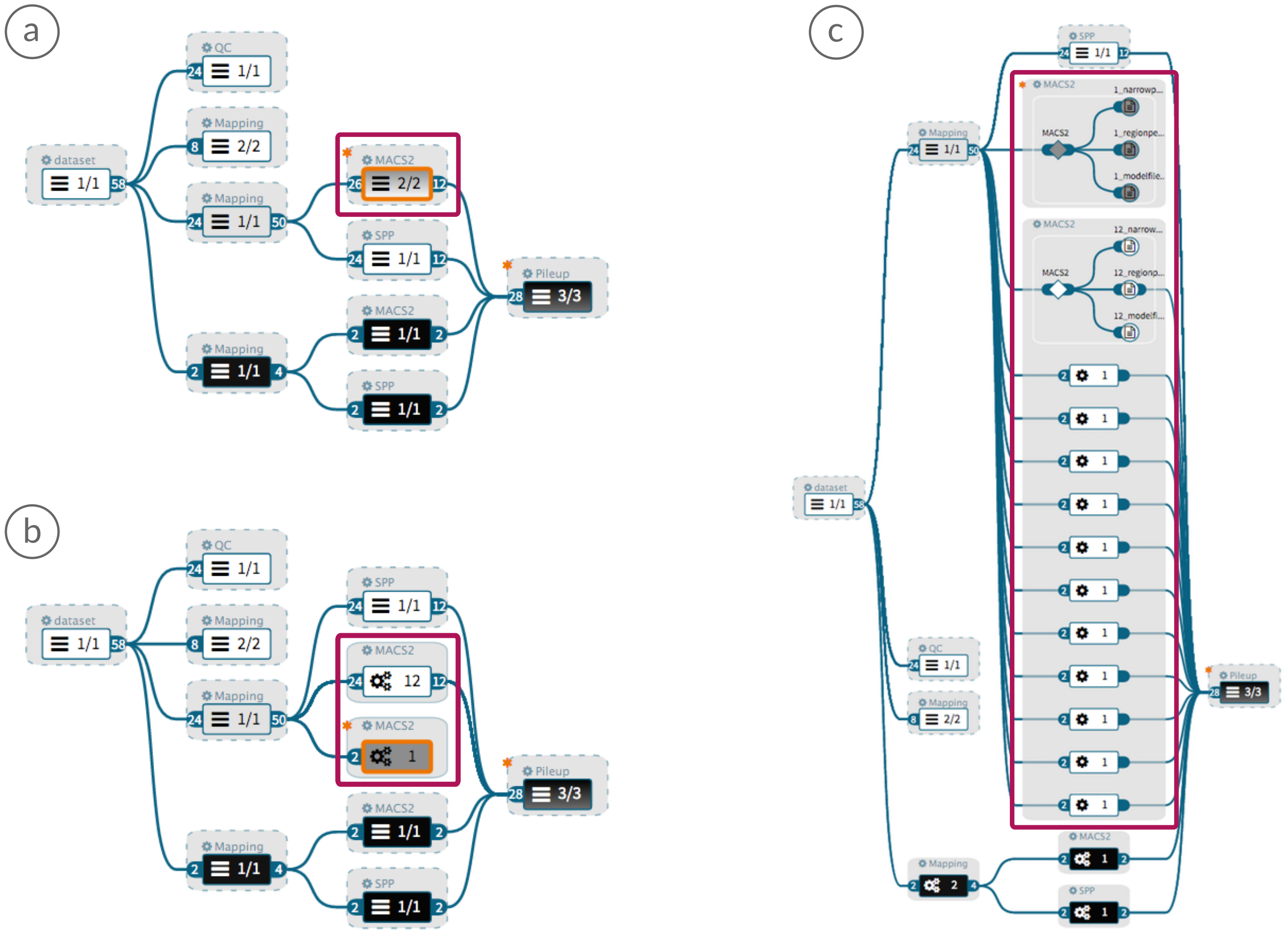

Figure S1: *Aggregated layer nodes that contain analyses with minor differences. The orange asterisk indicates that analyses that are using the same workflow template but with different parametrization (w.r.t. inputs, outputs, execution time, etc.) are aggregated into a single layer node. (a) The layer node is aggregated. (b) The layer node is expanded to reveal the two analyses, one with 12 analysis input groups and one with 1 analysis input group. (c) Further expansion reveals the workflow instances, which are only partially expanded in the bottom analysis.*

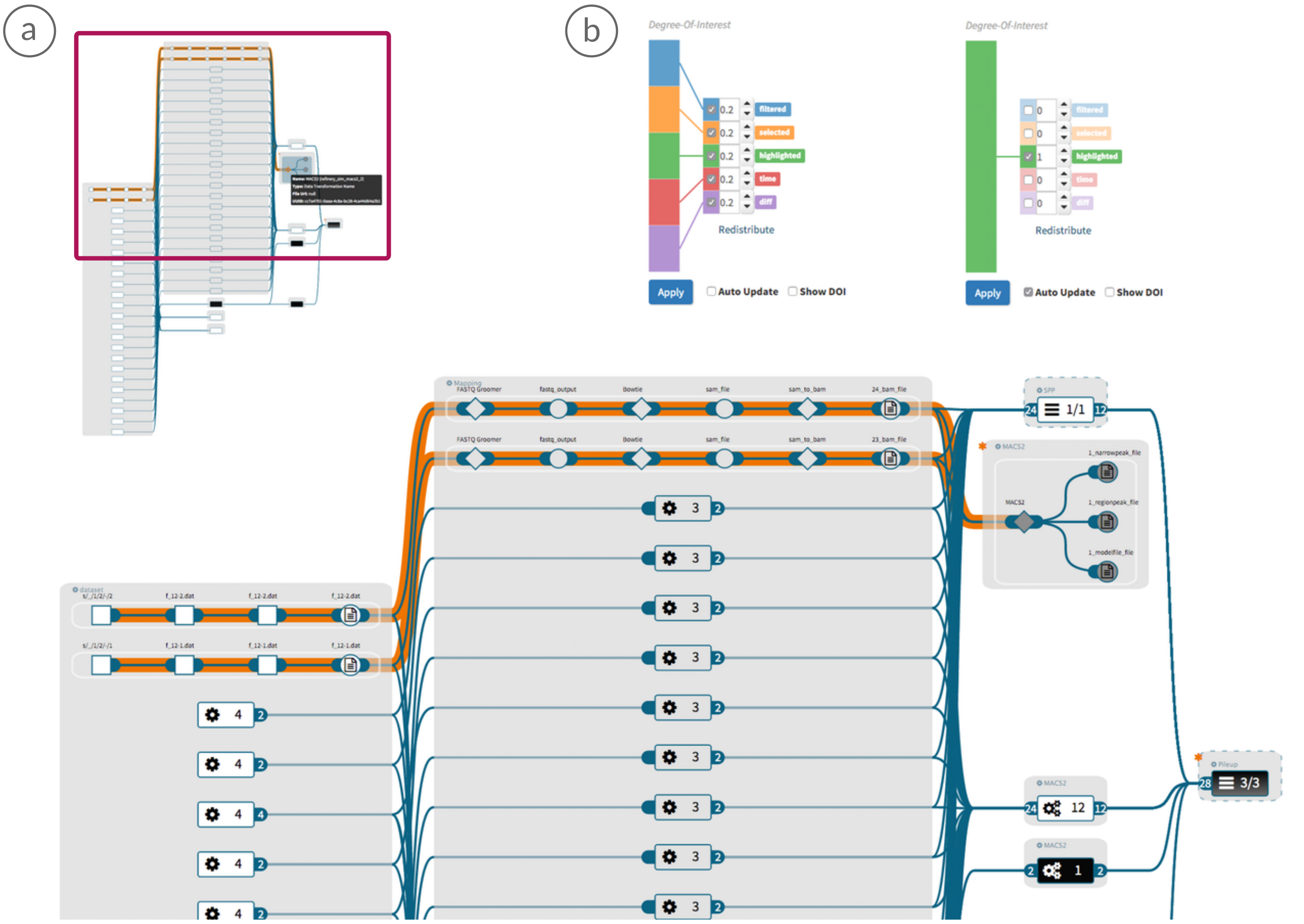

Figure S2: *Reviewing data provenance for the inputs of a selected tool. (a) The overview shows the whole provenance graph at different levels of aggregation. The nodes along the orange highlighting are fully expanded. The view at the bottom shows details of every data transformation step that was applied before the files were used as input for the MACS2 tool. (b) The DoI function controller set to default values (left) and to auto-expansion of selected paths (right). Auto-expansion is achieved by increasing the weight of the highlighted component to 1 (maximum) and reducing the weight of all other components to 0 (minimum). The Auto update option applies the DoI function immediately on every user interaction.*

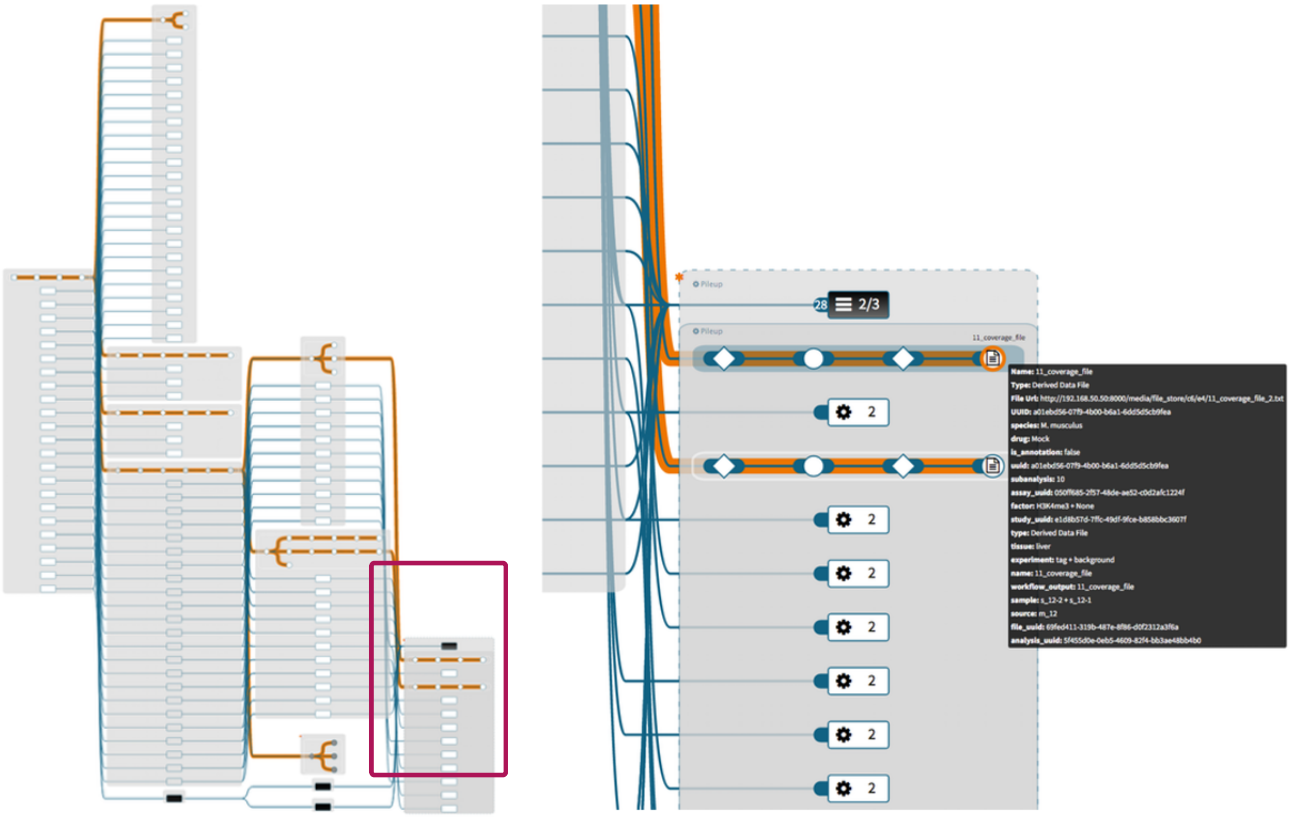

Figure S3: *Identifying results generated based on a selected raw data file. The overview on the left shows the paths from the selected raw data file to all derived results. Note that the semantic zoom removes labels and other details when there is not enough room to display them. The view on the right shows details about a Pileup workflow that generated some of the results derived from the selected raw data file. The expansion of detailed workflow level information is automatically limited to those nodes that are relevant for this task.*

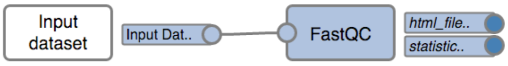

Figure S4: *Quality control workflow with 1 input and 2 outputs.*

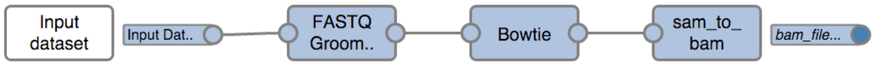

Figure S5: *Mapping workflow with 1 input and 1 output.*

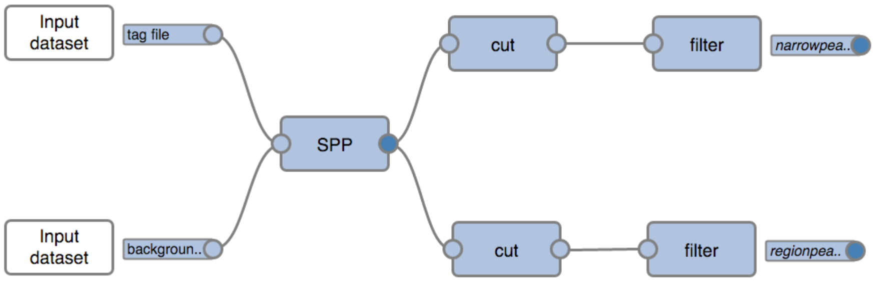

Figure S6: *SPP workflow with 2 paired inputs and 3 outputs.*

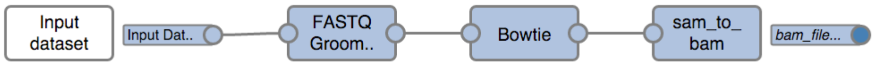

Figure S7: *MACS2 workflow 2 paired inputs and 3 outputs.*

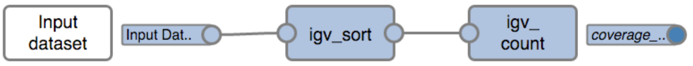

Figure S8: *Pileup workflow 1 input and 1 output.*
